# Multi-Ligand Interactions Shape Human Norovirus Persistence, Transmission, and Control in Food Matrices

**DOI:** 10.3390/v18070731

**Published:** 2026-07-01

**Authors:** Zilei Zhang, Junshan Gao, Yingyin Liao, Xuchong Zhao, Shumin Li, Danlei Liu, Liang Xue

**Affiliations:** 1 Department of Customs Inspection and Quarantine, Shanghai Customs University, Shanghai 201204, China; zhangzilei@shcc.edu.cn; 2 State Key Laboratory of Applied Microbiology Southern China, Institute of Microbiology, Guangdong Academy of Sciences, Guangzhou 510070, China; 3 Department of Microbiology, Li Ka Shing Faculty of Medicine, The University of Hong Kong, Hong Kong SAR, China; 4 Jinan Center for Disease Control and Prevention, Jinan 250118, China; 5 College of Veterinary Medicine, Jilin University, Changchun 130062, China; 6 Shanghai International Travel Healthcare Center, Shanghai Customs District P.R. China, Shanghai 200335, China

**Keywords:** human norovirus, foodborne transmission, multi-ligand interactions, risk assessment, precision intervention

## Abstract

Human norovirus (HuNoV) is the leading cause of foodborne viral gastroenteritis worldwide, yet its persistence in foods is still commonly interpreted through a simplified framework of contamination and residual survival. Accumulating evidence indicates that HuNoV persistence in food systems may be shaped by dynamic, genotype-dependent interactions with multiple classes of candidate ligands and retention mechanisms associated with hosts, food matrices, and microbiota. This review synthesizes current advances in the molecular basis and ecological consequences of these interactions, with emphasis on canonical and non-canonical glycans, HBGA-like substances, proteinaceous ligands, and bacterial surface or matrix-associated components. Structural, biophysical, and food-model studies collectively suggest that such factors may modulate capsid engagement, tissue retention, bioaccumulation, environmental stability, and, in some experimental systems, infectivity-related outcomes in representative matrices including leafy vegetables, bivalve mollusks, and bacteria-rich food environments. This multi-ligand perspective helps explain the matrix-dependent limitations of conventional washing, depuration, disinfection, and nucleic acid-based detection, as well as the frequent disconnect between measured viral signals and actual transmission risk. By linking molecular recognition to real food scenarios, this review highlights a shift from single-receptor and single-treatment perspectives toward mechanism-informed detection, risk assessment, and intervention strategies. A more integrated understanding of virus-ligand-matrix-microbiota interactions will be essential for improving the prediction and control of HuNoV foodborne transmission.

## 1. Introduction

### 1.1. Global Impact and Epidemiological Challenges

Human norovirus (HuNoV) is the primary etiology of non-bacterial acute gastroenteritis (AGE) worldwide, accounting for approximately 18% of all diarrheal cases across all age groups [1]. As a dominant foodborne pathogen, HuNoV is responsible for over 90% of non-bacterial gastroenteritis outbreaks globally [2,3]. Current estimates suggest that HuNoVs cause roughly 685 million infections annually [4], leading to 212,000 to 218,000 deaths, with nearly 99% of these fatalities occurring in low-to-middle-income countries [1]. Clinical manifestations typically include nausea, vomiting, and severe diarrhea, which are generally self-limiting within 1–3 days in healthy individuals [5]; however, they can progress to severe dehydration or chronic infection in pediatric, elderly, and immunocompromised populations [6].

The global socio-economic burden of HuNoV is profound, with annual societal costs estimated between $60 billion and $64 billion [4]. These figures include approximately $4.2 billion in direct healthcare costs and over $60 billion in wider societal losses [7]. Despite these staggering numbers, the true impact of HuNoV is likely underestimated, as many foodborne cases are self-managed without clinical confirmation or official reporting [8]. The epidemic potential of HuNoV is driven by its formidable biological characteristics: (1) an exceptionally low infectious dose, estimated at <100 copies or as few as 18–20 viral particles [9]; (2) high levels of viral shedding, reaching 10^6^ to 10^9^ particles per gram of feces during infection [10]; (3) efficient transmission via aerosols generated by vomiting [11,12]; and (4) extraordinary environmental resilience [13]. HuNoVs can withstand a wide pH range, temperatures of 60 °C for at least 30 min, and various common disinfectants including ethanol, chlorhexidine, and triclosan, allowing them to persist on surfaces and within food supply chains for several weeks [14,15].

### 1.2. Virion Architecture and Structural Basis of Host Interaction

HuNoVs are non-enveloped, positive-sense, single-stranded RNA viruses belonging to the *Norovirus* genus within the *Caliciviridae* family. The viral particles exhibit icosahedral symmetry (T = 3) with a diameter of approximately 25–40 nm [16,17]. The genome, spanning roughly 7.5–8.3 kb, typically contains three major open reading frames (ORFs). ORF1 encodes a polyprotein subsequently cleaved into six non-structural proteins, including the RNA-dependent RNA polymerase (RdRp) [18]. ORF2 and ORF3 encode the major capsid protein (VP1) and the minor structural protein (VP2), respectively. While VP1 is the primary structural component, recent evidence suggests that VP2 can form a portal-like assembly following receptor engagement, which serves as a channel for genome delivery during infection [17].

The HuNoV capsid consists of 180 VP1 units organized into 90 dimers. Structurally, VP1 is divided into the N-terminal shell (S) domain (residues 1–213), which forms the internal scaffold encapsulating the RNA, and the C-terminal protruding (P) domain (residues 222–539), which extends from the shell to form the characteristic arch-like protrusions [19]. The P domain is further subdivided into P1 and P2 subdomains, with the highly exposed P2 subdomain forming the apex of the capsid. The P2 subdomain is the most genetically variable region and serves as the primary site for host receptor recognition-most notably histo-blood group antigens (HBGAs)-and immune neutralization [20]. Recent structural studies highlight the conformational plasticity of the P domain, which can transition between “resting” and “rising” states, potentially influencing viral stability and ligand accessibility within different environmental or physiological conditions [19].

Noroviruses are currently classified into 10 genogroups (GI-GX), with 48 distinct genotypes based on the complete VP1 amino acid sequence [21]. HuNoVs predominantly belong to genogroups GI, GII, GIV, GVIII, and GIX. Although GII.4 has been the globally dominant genotype for decades [22], responsible for approximately 80% of outbreaks, the epidemiological landscape is dynamic [23]. New variants frequently emerge through antigenic drift and recombination, as evidenced by the recent rise of GII.17 Kawasaki 308 and GII.2 [P16] strains [24]. To accurately monitor these evolutionary shifts, a dual nomenclature system is employed, in which strains are classified by both their capsid genotype and RdRp P-type [21]. Thus, representative strains may be designated as GI.6 [P10], GII.4 Sydney [P16], or GII.17 [P17]. In the updated classification framework, noroviruses are divided into 48 confirmed capsid genotypes based on the amino acid sequence of the complete VP1 and 60 confirmed P-types based on partial nucleotide sequences of the RdRp region [21]. This extensive genetic diversity, coupled with the virus’s ability to recognize various glycans through its flexible P2 subdomain, facilitates its successful transmission across diverse and complex food matrices.

### 1.3. The Cultivation Bottleneck

The historical recalcitrance of HuNoVs to robust and standardized in vitro cultivation has fundamentally hindered the characterization of their infection mechanisms. While early attempts utilizing immortalized cell lines were largely unsuccessful, the development of B-cell culture systems provided initial evidence that enteric bacteria-derived HBGAs could facilitate replication [25], though these systems often lacked reproducibility and suffered from suboptimal yields [26,27]. A significant paradigm shift occurred with the advent of human intestinal enteroids (HIEs) [28], which successfully support the replication of multiple HuNoV genotypes, including GI.1, GII.3, GII.4, and GII.17, with subsequent studies further expanding the range of cultivatable GI and GII strains [28,29,30]. In these systems, productive replication is reflected by genotype- and culture-dependent increases in viral genome copy number, typically within the log10 range, with GII.4 strains often showing higher replication levels than several other tested genotypes [28,29,30]. This model confirmed the necessity of specific cofactors, such as bile acids and ceramides, for genotype-specific entry (e.g., GII.3) [31]. Nevertheless, HIEs remain technically laborious, prohibitively expensive, and constrained by significant strain-dependent success rates, which currently limits their suitability for high-throughput antiviral screening [29]. Consequently, zebrafish (Danio rerio) larvae have emerged as a promising high-throughput alternative, supporting the replication of multiple genotypes-including GI.7, GII.3, GII.4, and GII.6-without the prerequisite of bile acid supplementation [32]. Furthermore, the zebrafish model has been utilized to evaluate the efficacy of environmental interventions, such as the decay of viral infectivity following ultraviolet (UV) treatment [33].

To circumvent the challenges associated with infectious HuNoV, researchers have relied extensively on surrogate viruses and subviral particles. While biological surrogates like feline calicivirus (FCV) and murine norovirus (MNV) are cultivable [34,35], their reliance on distinct proteinaceous receptors (JAM-A and CD300lf, respectively) limits their ability to accurately recapitulate the glycan-dependent host interactions characteristic of HuNoV [36,37,38]. In contrast, virus-like particles (VLPs) and P-particles offer structurally authentic alternatives that preserve the antigenic landscape and HBGA-binding functionality of the native virion [18,39,40].

Beyond standard surrogates, specialized discovery platforms have been implemented to mine novel HuNoV ligands from complex food matrices. For instance, a bacterial cell surface display system has been successfully utilized to identify novel interaction partners in lettuce and oysters, including the hexasaccharide H2N2F2 and endogenous proteins such as oHSP70, oTNF, and oIFT [41,42,43]. These experimental approaches are increasingly augmented by cutting-edge biophysical and computational tools. The integration of AlphaFold3-based structural prediction with molecular dynamics (MD) simulations and Saturation Transfer Difference (STD) NMR spectroscopy enables the atomic-level resolution of the dynamic interplay between HuNoV capsids and diverse glycans [44,45]. Collectively, these transitions from traditional cultivation efforts to advanced bioengineering and in silico modeling provide the essential technical foundation for decoding the interactions between HuNoV and food matrix ligands.

### 1.4. Scope and Aim of This Review

Existing reviews have established important foundations for understanding HuNoV biology. Nevertheless, foodborne transmission occurs in complex matrix environments in which viral particles encounter diverse ligands, structural barriers, and microecological factors that together influence their physical state, persistence, recoverability, and susceptibility to control. In this context, this review provides a food-matrix-centered synthesis of the ligand classes and retention mechanisms currently implicated in HuNoV persistence across representative food systems, including leafy vegetables, bivalve mollusks, and bacteria-rich microenvironments. Particular attention is given to canonical and non-canonical glycans, HBGA-like substances, proteinaceous ligands, and bacterial envelope- or matrix-associated components, with emphasis on how these matrix-specific interactions may shape viral attachment, internalization, bioaccumulation, environmental stability, detection performance, and intervention outcomes, while also highlighting the key evidence gaps that remain relevant to risk assessment and mechanism-informed food safety control.

## 2. Molecular Basis of Norovirus-Ligand Interactions

### 2.1. Genetic Polymorphism and Biosynthetic Diversity of HBGAs

Susceptibility to HuNoV infection is closely linked to the host’s HBGAs profile [46]. HBGAs are a structurally diverse class of fucosylated glycans, widely distributed on red blood cells, intestinal epithelial cells, and in biological fluids such as saliva and breast milk [47,48]. Their biosynthesis is tightly regulated by fucosyltransferases (FUTs), among which FUT2 mediates the addition of α1,2-fucose to the terminal β-galactose of Type 1 or Type 2 precursors to form the H antigen [48]. Genetic polymorphisms in FUT2, particularly loss-of-function mutations, give rise to the non-secretor phenotype [24], characterized by the absence of α1,2-fucosylated HBGAs and associated with strong resistance to major epidemic genotypes, including GI.1, GII.4, and GII.17. Together, these early findings established the conceptual basis for understanding secretor-dependent susceptibility and provided an essential foundation for subsequent studies on genotype-specific ligand recognition, host range, and viral adaptation [49,50].

#### 2.1.1. Genotype-Specific Recognition Patterns

HuNoV particles engage HBGAs through binding pockets (BPs) located at the dimer interface of the protruding (P) domain [51]. Through divergent evolution, GI and GII genogroups have developed distinct recognition patterns. At the structural level, these genotype-dependent differences are determined by the architecture of the binding pocket at the P-domain dimer interface rather than by a simple preference for a single terminal alone. Crystallographic and affinity studies have shown that GI pockets often accommodate galactose-containing motifs through a combination of hydrogen bonding and shape complementarity, whereas in many GII strains, α-fucose serves as a major anchoring residue that organizes the surrounding interaction network within the pocket [51,52,53]. Variations in neighboring loops and side-chain orientation further modulate pocket depth, steric accessibility, and the contribution of local hydrophobic contacts, thereby broadening or restricting ligand recognition across genotypes [51,52,53,54,55]. In addition, ligand engagement occurs within a conformationally dynamic P-domain dimer, so local flexibility may influence pocket exposure, mechanical stability, and the capacity of closely related strains to accommodate structurally distinct glycans [19]. These structural features help explain why HBGA recognition is both genotype-dependent and adaptable, and they provide a mechanistic basis for considering how HBGA-like or food-derived glycans may be differentially accommodated in later food-matrix contexts. Biophysical studies have highlighted the dynamic nature of these interactions; for example, atomic force microscopy (AFM) measurements have shown that fucose binding significantly alters the mechanical stability of GII.10 and GII.17 virus-like particles [19]. Furthermore, comparative analyses across all nine GI genotypes have identified the Lewis b (Leb) antigen as a shared ligand, suggesting a potential role in cross-species transmission [56].

#### 2.1.2. Evolutionary Dynamics

HuNoVs continuously reshape the architecture of their binding pockets to facilitate immune evasion and expand host susceptibility [24,53]. From a structural perspective, even subtle remodeling of pocket-defining loops or side-chain orientation can alter hydrogen-bond geometry, steric accessibility, and dimer flexibility, thereby shifting ligand breadth and binding efficiency during the emergence of new variants [51,52,53,54,55]. The GII.4 genotype, which has dominated global epidemics for decades, exhibits broad recognition of ABH and Lewis antigens across secretor populations, a feature that underpins its sustained prevalence [57,58]. Similarly, emerging variants such as GII.17 Kawasaki 308 have acquired mutations within the P domain (e.g., V444Y) that enhance α1,2-fucose binding, contributing to their epidemic potential [24]. These observations highlight the dynamic nature of ligand engagement, whereby subtle structural remodeling of the P domain enables shifts in binding affinity and host range.

### 2.2. Expanding Ligand Recognition Beyond Canonical HBGAs

#### 2.2.1. Diversification of the Glycan Recognition Spectrum

Although HBGAs represent the best-characterized attachment factors for HuNoVs, increasing evidence indicates that these viruses can engage a broader repertoire of glycan ligands [59]. Heparan sulfate has been identified as an attachment factor on host cell surfaces for GII strains [60], while sialylated glycans have demonstrated comparable binding affinities to several HuNoV genotypes [54,61,62]. In addition, small molecules such as citrate can occupy the HBGAs binding site, likely through structural mimicry of fucose, thereby modulating ligand engagement [55]. Collectively, these findings suggest that HuNoV attachment is not restricted to canonical HBGAs but instead reflects a flexible and context-dependent glycan recognition strategy.

#### 2.2.2. Evolutionary Shifts in Glycan Specificity

The high mutation rate of the HuNoV genome drives continuous adaptation of its glycan-binding interfaces, enabling certain variants to reduce their dependence on canonical HBGAs [53]. Structural analyses have shown that conformational rearrangements in surface loops of the P domain can reshape the BPs, altering ligand accessibility and specificity. Such adaptations allow the virus to exploit alternative glycans present in the intestinal environment, thereby expanding its ecological niche. Notably, similar shifts in glycan usage have been observed across multiple genotypes [63], underscoring the evolutionary plasticity of HuNoV receptor engagement.

#### 2.2.3. Food-Derived Glycan Ligands and Environmental Persistence

Collectively, the expanding spectrum of HuNoV glycan recognition, coupled with the structural adaptability of the P domain, defines a highly flexible and context-dependent mode of ligand engagement. In this framework, glycan interactions are not restricted to host-derived HBGAs but may extend to structurally related glycans encountered in environmental and food-associated settings [41]. Emerging evidence indicates that such interactions, particularly with core-fucosylated glycans, can occur in vitro and may contribute to the stabilization of viral particles [44]. Rather than functioning as primary receptors, these glycans are more likely to act as environmental reservoirs or stabilizing factors, thereby facilitating foodborne transmission and reducing susceptibility to decontamination processes. This conceptual extension provides a mechanistic basis for understanding HuNoV persistence in food matrices and sets the stage for examining virus–food interactions in real-world contexts.

## 3. Interactions Within Complex Food Matrices

**Figure 1:**
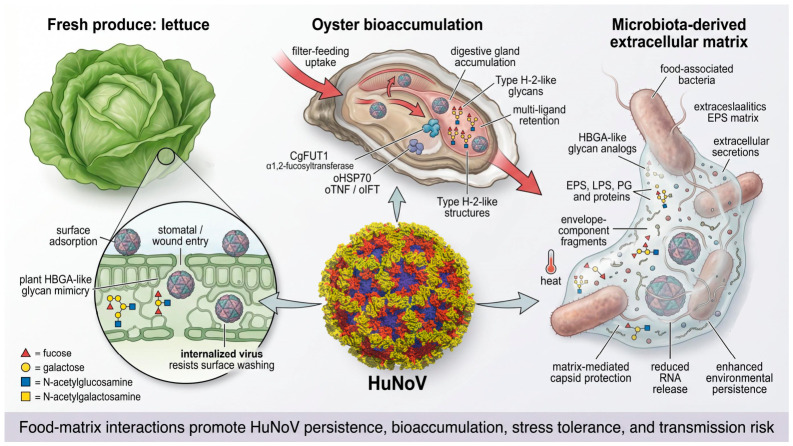
Multi-ligand interactions associated with HuNoV persistence across representative food matrices. The schematic representation of the HuNoV virion was adapted with reference to Vinjé et al. [18]. Codex (GPT-5.4) was used to assist in generating prompts for Figure 1, and the figure image was generated using the online tool PicDoc (https://www.picdoc.ai/).

Building upon the glycan-mediated recognition framework and its expanding diversity, food matrices are increasingly recognized as active modulators of HuNoV stability, accumulation, and transmission. Rather than serving as passive carriers, these complex environments provide a spectrum of physicochemical conditions and biological ligands that influence viral persistence and infectivity. Accordingly, the following sections examine HuNoV interactions in three representative systems: fresh produce (lettuce), bivalve mollusks (oysters), and commensal or food-associated bacteria. Figure 1 summarizes how matrix-specific ligands and microecological components may influence HuNoV attachment, internalization, bioaccumulation, and environmental persistence in representative food systems. At present, the available direct food-matrix interaction data remain concentrated primarily in GI.1 and GII.4, while comparable evidence for other clinically important genotypes, including GII.2, GII.3, and GII.6, remains comparatively limited.

### 3.1. Adhesion and Internalization of HuNoV in Fresh Produce

Following the overarching role of foodborne transmission in HuNoV epidemiology, fresh produce-and lettuce in particular-represents one of the most prominent vehicles implicated in large-scale outbreaks [1]. This is largely attributable to its frequent consumption in raw or minimally processed forms, which preserves viral infectivity at the point of ingestion [64,65,66].

In the context of previously described glycan-mediated interactions, including canonical HBGAs and related ligands [67], HuNoV attachment to lettuce likely involves both non-specific physicochemical forces and more selective recognition of HBGA-like or structurally analogous glycans. Against this backdrop, the interaction between HuNoVs and lettuce can be understood across multiple levels, encompassing surface adsorption, tissue internalization, and molecular-scale glycan recognition.

#### 3.1.1. Physicochemical Interactions and Internalization Dynamics

Within lettuce matrices, the persistence of HuNoVs is governed by the combined effects of physicochemical interactions and biological entry processes. Initial attachment is largely mediated by non-specific forces, among which electrostatic interactions play a dominant role [68]; increasing ionic strength (e.g., via NaCl supplementation) has been shown to attenuate viral adsorption.

More critically, HuNoVs can internalize into plant tissues through root uptake, stomatal openings, or damaged leaf surfaces, thereby circumventing external barriers [69,70]. Experimental models of waterborne contamination have demonstrated that viral RNA can persist within internal tissues for extended periods (up to 14 days). This internalized state substantially limits the effectiveness of surface decontamination strategies [71], including chlorine-based washing, and represents a persistent risk for foodborne transmission.

#### 3.1.2. Genotype-Specific Recognition in Plant Matrices

Beyond non-specific adsorption, HuNoV–lettuce interactions exhibit pronounced genotype-dependent recognition patterns, consistent with the flexible glycan engagement strategies described above. Distinct binding preferences have been observed across genotypes: GI.1 strains tend to associate with proteinaceous components on the leaf surface, whereas GII.4 strains preferentially interact with polysaccharide constituents, including HBGA-like structures within the plant cell wall [3,72].

Importantly, pre-incubation with HBGA-containing ligands (e.g., porcine gastric mucin) does not fully inhibit viral attachment, indicating that plant matrices harbor diverse receptor analogs that function independently of canonical human HBGAs [73]. This multiplicity of binding modes provides a mechanistic basis for genotype-dependent differences in environmental persistence and outbreak patterns.

#### 3.1.3. Molecular Characterization of Plant-Derived Glycan Ligands

At the molecular level, recent advances have begun to identify candidate glycan determinants that may contribute to HuNoV–plant interactions. Using a bacterial cell surface display system, a representative core-fucosylated hexasaccharide (H_2_N_2_F_2_) has been identified from lettuce matrices [41]. This glycan contains antigenic features characteristic of A, H, and Lewis a HBGAs within a branched structure, suggesting that it may mimic host-associated ligands.

Integrative analyses combining structural modeling and molecular dynamics simulations further suggest that H_2_N_2_F_2_ can occupy the canonical binding pockets of several globally prevalent genotypes, including GII.3, GII.4, and GII.6 [44]. These observations suggest that food-derived glycan recognition may extend beyond the most intensively studied GI.1 and GII.4 strains, although direct validation in intact food matrices remains limited for most non-dominant genotypes. These findings support the plausibility of glycan-mediated HuNoV–plant interactions at the molecular level. Despite this, the functional contribution of H_2_N_2_F_2_ to viral persistence in intact plant tissues remains to be validated directly, and its proposed role in fresh produce-associated persistence should therefore be interpreted with appropriate caution.

### 3.2. Multi-Ligand Contributions to HuNoV Bioaccumulation in Bivalve Mollusks

Complementing the role of fresh produce in foodborne transmission, bivalve mollusks-particularly oysters-represent another major vehicle for HuNoV outbreaks, especially in regions where raw consumption is common [74,75]. Unlike plant matrices, oysters actively concentrate viral particles from contaminated waters through filter-feeding, leading to efficient bioaccumulation within their tissues.

In light of the glycan-mediated recognition mechanisms discussed above, HuNoV-oyster interactions are characterized not only by HBGA-like glycans but also by additional ligand systems that facilitate viral retention. Consequently, viral persistence in oysters arises from coordinated processes involving tissue-specific accumulation, endogenous ligand biosynthesis, and multi-modal molecular recognition.

#### 3.2.1. Spatial Distribution Patterns and Bioaccumulation Dynamics

Bivalve mollusks such as oysters efficiently concentrate HuNoVs from contaminated aquatic environments through filter-feeding [67]. High-resolution analyses using controlled contamination models have revealed that viral distribution within oyster tissues follows a distinct spatial pattern. Following uptake, viral particles are transported along the digestive pathway, with peak accumulation occurring in the digestive gland, where detection rates can reach up to 95.83% [76]. These earlier observations established that shellfish are not simply passive vehicles of contamination, but biologically selective matrices in which HuNoVs can undergo tissue-specific accumulation and retention [77,78,79], thereby laying the groundwork for subsequent studies on glycan- and ligand-associated bioaccumulation.

This bioaccumulation process exhibits marked genotype-dependent differences, with GI.1 strains demonstrating higher enrichment efficiency and prolonged persistence compared to GII.4 strains [80,81]. The pronounced tissue tropism and sequestration of HuNoVs within the digestive gland substantially limit the effectiveness of conventional depuration processes, thereby posing a persistent risk to food safety.

#### 3.2.2. HBGA-like Substances and Endogenous Biosynthesis

The high-affinity retention of HuNoVs in oysters has been largely attributed to the presence of HBGA-like glycans within molluskan tissues. Glycan structures recognized by anti-A and anti-H antibodies are widely distributed throughout the gastrointestinal tract, particularly within the digestive gland [82,83].

Mechanistically, oysters possess the endogenous capacity to synthesize such receptor analogs. The identification and functional characterization of the fucosyltransferase gene CgFUT1 in the Pacific oyster (Crassostrea gigas) demonstrated α1,2-fucosyltransferase activity, enabling the synthesis of Type H-2-like structures [84]. Notably, the expression of CgFUT1 is highest in the digestive gland, consistent with the primary sites of viral accumulation. These findings provide a structural and biochemical basis for the selective enrichment of HuNoVs in oyster tissues.

#### 3.2.3. Potential Contributions of Glycan and Proteinaceous Ligands

While HBGA-like glycans likely represent an important basis for viral retention in oysters, they may not fully explain the genotype-specific patterns of HuNoV bioaccumulation observed in shellfish. Additional evidence has suggested that candidate proteinaceous ligands may also be involved in this process.

Proteomic analyses have identified several host proteins with reported HuNoV-binding activity, including oyster heat shock protein 70 (oHSP70), which has been associated with GII.4 binding and has been detected in the digestive gland and gill tissues [42]. For GI.1 strains, additional candidates such as oyster tumor necrosis factor (oTNF) and intraflagellar transport protein (oIFT) have also been reported to exhibit binding capacity [43]. These observations suggest that proteinaceous ligands may contribute to genotype-associated retention patterns in oysters. However, their physiological accessibility and functional contribution to viral sequestration in intact oyster tissues remain to be further clarified.

Taken together, current findings suggest that HuNoV bioaccumulation in oysters may involve multiple candidate retention determinants, including both HBGA-like glycans and proteinaceous ligands. At present, the relative contribution of these factors to in vivo viral enrichment and persistence remains incompletely resolved.

### 3.3. Microbiota-Mediated Stabilization of HuNoV

In addition to plant and molluscan matrices, food-associated microbiota constitute a dynamic biological interface that further modulates HuNoV persistence and transmission. These microbial communities participate in virus–matrix interactions through surface-exposed glycans, envelope components, and biofilm-associated structures. Earlier work on norovirus–bacterium interactions helped expand the field from a predominantly host-glycan-centered framework toward a broader view that also considers microbiota-associated effects on viral attachment, stability, and infectivity [85,86]. Building upon the glycan-mediated recognition framework described above, food-associated microbiota can facilitate viral attachment, stabilize capsid architecture, and enhance tolerance to environmental stress, thereby expanding the functional landscape of HuNoV–food matrix interactions. Consequently, HuNoV persistence in complex food environments is increasingly recognized as a microbiota-assisted process involving direct binding, structural stabilization, and potential infectivity enhancement.

In addition to plant and molluscan matrices, food-associated microbiota represent a dynamic biological interface that may further influence HuNoV persistence and transmission. These microbial communities may participate in virus–matrix interactions through surface-exposed glycans, envelope components, and biofilm-associated structures. Available studies suggest that microbiota-associated factors may contribute to viral attachment, capsid stabilization, and tolerance to environmental stress, thereby extending the range of matrix-associated processes potentially relevant to HuNoV persistence in foods. At present, much of this evidence derives from in vitro systems, VLP-based assays, or surrogate viruses, and direct validation in authentic foodborne HuNoV contexts remains limited.

#### 3.3.1. Direct Engagement Mediated by Bacterial Surface Glycan Analogs

Interactions between HuNoVs and complex food matrices are frequently mediated by bacteria residing within these environments. A wide range of enteric and food-associated bacteria have been shown to express glycan epitopes that structurally mimic mammalian attachment factors, including Type A, B, and H HBGA analogs as well as sialylated glycans [87].

A representative example is *Enterobacter* sp. SENG-6, an intestinal isolate whose extracellular polymeric substances contain Type A HBGA-like moieties capable of specifically capturing HuNoV virus-like particles [88]. More broadly, members of the *Enterobacteriaceae* [89], certain probiotic strains [90], and bacteria colonizing produce surfaces can synthesize such glycan analogs through endogenous pathways [91]. These observations suggest that bacteria-mediated association may contribute to viral attachment to food matrices and may, under some conditions, influence local protection from external stressors.

#### 3.3.2. Stabilization of Viral Architecture by Bacterial Envelope Components

Beyond mediating attachment, bacteria and their envelope constituents have been reported to contribute to the preservation of capsid integrity under certain experimental conditions. Components such as lipopolysaccharides (LPS), exopolysaccharides (EPS), and peptidoglycan (PG) have been associated with increased viral tolerance to environmental stress in in vitro systems, VLP-based assays, or surrogate-virus models [71,92].

Associations with HBGA-expressing bacteria have been reported to preserve capsid integrity and ligand-binding capacity under conditions of thermal stress, ultraviolet exposure, and chlorine-based disinfection. In particular, LPS from an *E. coli* O111:B4 has been shown to interact with the viral capsid and to reduce premature RNA release under adverse conditions [71]. These observations support the plausibility of component-mediated stabilization as one factor that may contribute to HuNoV persistence, although direct evidence from infectious HuNoV in authentic food matrices remains limited.

#### 3.3.3. Microbiota-Driven Potentiation of Viral Infectivity

In addition to effects on stability, microbiota-associated factors may also influence HuNoV infectivity under certain experimental conditions. In vitro studies have demonstrated that the presence of HBGA-producing bacteria, such as *Enterobacter cloacae*, can facilitate viral infection in B cells [25]. Furthermore, bacterial lysates and surface structures have been reported to augment viral entry in experimental systems [92]. Insights from surrogate models, including murine norovirus, further suggest that bacterial features such as flagella, together with electrostatic interactions, may contribute to efficient virus-bacterium association. These interactions may alter the physicochemical properties of the virion and may further influence infection outcomes by modulating local host responses. However, direct evidence for such effects on infectious HuNoV in authentic foodborne contexts remains limited.

### 3.4. An Integrated Food-Matrix Perspective on HuNoV Persistence

Across representative food systems, HuNoV persistence appears to be associated with matrix-specific repertoires of candidate ligands and retention pathways rather than a single universal mode of attachment. In fresh produce, surface adsorption, tissue internalization, and plant-derived glycans may all contribute to viral persistence; in oysters, tissue-specific accumulation is linked to filter-feeding-mediated uptake, HBGA-like glycans, and additional proteinaceous ligands; and in bacteria-rich food environments, glycan analogs, envelope components, and extracellular matrices may further influence viral retention and stability. Taken together, these observations suggest that HuNoV may exist in foods in multiple physical and biological states shaped by matrix composition, genotype, and microecological context. Such diversity is important because different modes of retention are likely to influence viral recovery, detection performance, and susceptibility to control in distinct ways, thereby providing an integrated framework for understanding HuNoV persistence in food systems.

## 4. Implications for Risk Control and Intervention Strategies

The preceding sections have delineated the persistence mechanisms of HuNoVs in food systems from three interconnected perspectives: molecular recognition, food matrix interactions, and microbiota-associated effects. HBGAs and HBGA-like glycans provide both canonical and non-canonical binding sites for HuNoVs, while plant-derived H2N2F2 hexasaccharides, H-type glycans in oyster tissues, and proteinaceous ligands such as oHSP70, oTNF, and oIFT further expand the repertoire of food matrix ligands available for viral engagement. In parallel, bacterial envelope components, including lipopolysaccharides (LPS), exopolysaccharides (EPS), and peptidoglycan (PG), can enhance viral attachment, capsid stability, and tolerance to environmental stress. Collectively, these findings emphasize that HuNoVs food safety control should gradually move beyond a narrow focus on viral inactivation and incorporate a more precise understanding of the physical and biological states in which viral particles persist within real food matrices.

### 4.1. Limitations of Conventional Decontamination Strategies

Chlorination, ozone treatment, organic acid washing, thermal processing, and shellfish depuration have long been applied for viral contamination control along the food supply chain [93]. Leafy vegetables and soft fruits are commonly treated by washing, chlorine-based disinfectants, or organic acids to reduce surface contamination, whereas shellfish products rely primarily on depuration to promote the elimination of contaminants. However, as discussed in Section 3, HuNoVs can establish multiple stable states within food systems through tissue internalization, high-affinity ligand anchoring, and microbial-complex-mediated protection. Conventional treatments mainly target viral particles that are accessible, elutable, or externally exposed, which limits their efficacy against internalized virions, tissue-sequestered particles, and bacteria-associated viral complexes.

#### 4.1.1. Restrictions of Viral Removal

In fresh produce, the residual risk of HuNoVs is closely associated with both surface adsorption and tissue internalization. As described in Section 3.1, HuNoVs can adsorb to lettuce surfaces through electrostatic interactions and may further enter plant tissues through root uptake, stomatal openings, or damaged surfaces. Hydroponic contamination models have shown that viral RNA can remain detectable within internal lettuce tissues for up to 14 days [69]. Under such conditions, surface washing or chlorine treatment may reduce externally exposed viral particles, while exerting limited effects on virions that have already penetrated internal tissues.

Specific adsorption within plant matrices further increases the difficulty of viral removal. Core-fucosylated glycans of plant origin, such as the H2N2F2 hexasaccharide, can stably occupy the capsid binding pockets of multiple HuNoV genotypes with a high-affinity anchored state [41]. This stable molecular engagement can constrain viral release and reduce the efficacy of conventional washing, short-term chemical disinfection, and routine elution procedures.

In shellfish systems, the major limitation arises from tissue-specific enrichment and multi-ligand binding. Filter-feeding bivalves such as oysters concentrate HuNoVs from contaminated water, after which viral particles move along the digestive tract and accumulate at high levels in the digestive gland. Spatial distribution studies have reported detection rates of up to 95.83% in the digestive gland [76]. This enrichment is closely associated with HBGA-like glycans and proteinaceous ligands present in oyster tissues. This multiple ligand-associated retention factors limits the ability of conventional depuration to remove high-affinity bound virions, and the stronger retention capacity of GI strains further complicates risk control in shellfish products [43].

Food-associated bacteria provide another protective micro-environment. HuNoVs can form virus–bacteria complexes with bacteria expressing HBGA-like epitopes or with bacterial envelope components. LPS, EPS, and PG can stabilize the viral capsid and reduce premature RNA release under adverse environmental conditions [92]. Experimental evidence has shown that PG can increase viral survival by approximately 0.14 and 1.7 log PFU/mL under 200 ppm chlorine treatment and 60 °C thermal stress, respectively [71]. Bacterial biofilms may also reduce contact efficiency between disinfectants and viral particles, leading to lower inactivation efficacy in real food systems than that observed in simplified experimental settings.

#### 4.1.2. Transition from Treatment Intensity to Mechanism-Matched Control

The evidence summarized above indicates that HuNoV decontamination efficacy is jointly determined by treatment conditions and the physical state of the virus within the matrix. Simply increasing disinfectant concentration, extending depuration duration, or intensifying thermal treatment may offer limited additional benefit when viral particles are internalized, high-affinity-anchored, or protected by bacterial structures. Such approaches are also constrained by food quality, sensory properties, and processing feasibility. A more rational control framework should incorporate matrix-specific viral attachment modes, tissue distribution patterns, ligand engagement, and microecological protection into treatment design. For fresh produce, interventions should account for both surface-associated and internalized particles. For shellfish, depuration conditions should be optimized in relation to digestive-gland sequestration. In microbially complex environments, antibiofilm or matrix-disrupting pretreatments may be required to improve the accessibility of disinfectants to viral particles.

### 4.2. Mechanism-Informed Precision Intervention Strategies

Section 4.1 highlights the practical boundaries of conventional decontamination strategies in complex food systems. As the mechanisms underlying HuNoV–ligand interactions become increasingly clear, several intervention concepts can be proposed at different control points, including blocking ligand recognition during early attachment, weakening high-affinity retention within tissues or matrices, disrupting microbiota-associated protective effects, and designing broad-spectrum blocking molecules guided by structural biology.

#### 4.2.1. Blocking Ligand-Mediated Initial Attachment

The engagement between HuNoVs and HBGAs, HBGA-like glycans, or food-derived ligands represents an important step for viral persistence in food systems. Therefore, the use of soluble ligands or competitive molecules to occupy the binding sites within the viral P domain has been explored as a potential strategy for reducing initial attachment efficiency. Bovine colostrum is one representative example at the proof-of-concept level [94]. Studies have shown that bovine colostrum contains high-molecular-weight, multivalent glycoproteins that mimic human HBGA epitopes and bind the HuNoV P domain. At a dilution of 1:80, bovine colostrum can achieve more than 50% inhibition of HuNoV-HBGA binding in experimental systems, suggesting that food-derived natural glycoproteins may have potential as candidate blocking agents.

Nucleic acid aptamers represent another class of candidate blocking agents under active investigation [95,96]. Aptamers such as AP4-2 and AP17-4 have been reported to block the binding of GII.4 and GII.17 VLPs to porcine gastric mucin (PGM) and to inhibit internalization-related processes in cell-based experimental models [96].

#### 4.2.2. Weakening Tissue Anchoring and Matrix Retention

For HuNoVs that have already entered food matrices, precision intervention should focus on promoting viral dissociation from high-affinity binding states. Shellfish represent a particularly relevant application scenario for this strategy. In oyster digestive glands, HBGA-like glycans and candidate proteinaceous ligands such as oHSP70, oTNF, and oIFT may contribute to viral enrichment [42,43], and may help explain why conventional depuration does not always fully disrupt tissue-associated binding. Future studies may therefore explore whether blockers targeting candidate proteinaceous ligands or optimization of depuration parameters, such as temperature, salinity, pH, and metabolic state, can improve viral release from binding sites within the digestive gland. At present, these concepts remain exploratory and require validation under realistic shellfish-processing conditions.

Genotype-specific differences should also be incorporated into shellfish depuration design. Existing studies indicate that strains such as GI.1 may exhibit higher enrichment efficiency and longer retention times in shellfish than GII.4 [43]. Uniform depuration durations and conditions may therefore underestimate the residual risk posed by highly retained genotypes. Stratified depuration strategies based on genotype, tissue distribution, and binding strength may improve the precision of risk control for shellfish products.

#### 4.2.3. Disrupting Microbiota-Associated Protective Effects

Food-associated microbiota may influence HuNoV persistence through at least two general pathways-by providing binding structures such as HBGA-like or sialylated glycans that may facilitate viral attachment [91], and by stabilizing the viral capsid through LPS, EPS, and PG-and biofilm structures may represent a relevant target for future control strategies [92]. Accordingly, microbiota-associated protective structures may represent a relevant target for future control strategies. Antibiofilm treatments, EPS-degrading enzymes, peptidoglycan-disrupting agents, or pretreatments combined with chlorine, ultraviolet irradiation, or pulsed light may increase viral exposure and improve subsequent inactivation.

This concept is particularly relevant for food-processing environments and food-contact surfaces. When viral particles are embedded within biofilms or complexed with bacterial components, effective contact with conventional disinfectants is restricted. Disrupting the biofilm matrix before physical or chemical inactivation may therefore be more effective than simply increasing disinfectant intensity. In food systems with complex microecological structures, modulating the abundance of HBGA-like-expressing bacterial populations may also weaken virus–bacteria complex formation and reduce HuNoV stability throughout the supply chain.

#### 4.2.4. Broad-Spectrum Inhibitor Design Considering Conformational Flexibility and Genotypic Diversity

The high genetic diversity and structural plasticity of HuNoVs impose substantial challenges for the design of broad-spectrum blockers. As discussed in Section 2, GI and GII strains differ in their glycan recognition patterns, while prevalent strains such as GII.4 and GII.17 can alter binding pocket architecture and ligand affinity through mutations in the P domain [24,57]. NMR and structural studies have shown that ligand binding can modify the mechanical stability of viral particles [44,45], indicating that the capsid exists in a dynamically regulated conformational state. Inhibitor designs based exclusively on a single static structure may therefore have limited coverage.

Small-molecule studies further illustrate this challenge. For example, the reduced efficacy of rupintrivir against GII viruses has been associated with the H-G mutation in the BII-CII loop of the viral protease and the consequent reduction in binding pocket stability [97]. Future development of broad-spectrum inhibitors may benefit from integrating AlphaFold3-based structural prediction, molecular dynamics simulations, STD-NMR, AFM, and high-throughput screening to identify relatively conserved binding interfaces or key conformational transition processes across genotypes. However, such inhibitor concepts remain at an early stage of basic research, and substantial challenges remain before translation to food applications, including formulation, matrix compatibility, manufacturing scalability, cost, and food safety regulatory evaluation.

### 4.3. Key Challenges in Detection and Risk Assessment

As discussed above, HuNoV may persist in complex food matrices through multiple ligand-associated retention states, including surface adsorption, tissue internalization, high-affinity glycan binding, and microbiota-associated protection. These matrix-dependent states can in turn influence viral recovery, signal interpretation, and the assessment of foodborne transmission risk. Current foodborne virus detection largely relies on standardized methods such as ISO 15216-1:2017/Amd 1:2021 [98], which assess contamination levels through sample processing, viral elution, concentration, nucleic acid extraction, and RT-qPCR quantification. This framework provides a unified basis for HuNoV monitoring across the food chain, yet its outputs are influenced by viral recovery efficiency, matrix distribution, particle integrity, and the microecological environment. When viral particles are strongly ligand-bound, internalized within tissues, or protected within bacteria-associated complexes, the relationship between nucleic acid signals, actual contamination levels, and infection risk becomes substantially more complex.

#### 4.3.1. Recovery Bias Driven by High-Affinity Binding

For soft fruits and leafy vegetables, ISO 15216 generally involves chopping, elution, and PEG precipitation, with the key assumption that viral particles can be efficiently released from the sample matrix into the elution buffer [98]. Computational biology studies suggest that core-fucosylated glycans in lettuce cell walls, such as the H2N2F2 hexasaccharide, may engage the binding pockets of the HuNoV capsid [44]. This high-affinity molecular anchoring can make complete elution difficult using conventional buffers, even when viral particles are located on the plant surface. If virions have entered internal plant tissues, recovery becomes even more challenging. This mechanism-driven reduction in elution efficiency can directly decrease nucleic acid recovery and lead to underestimation of actual contamination levels.

Therefore, low detection rates or low viral loads in leafy vegetables should be interpreted in relation to extraction efficiency. Insufficient elution and reduced recovery may result in systematic underestimation of the true level of contamination.

#### 4.3.2. Interpretive Challenges Arising from Sampling Representativeness and Genotypic Differences

For shellfish testing, ISO 15216 designates the digestive gland as the primary target tissue for viral extraction and uses procedures such as Proteinase K digestion [98]. This specification aligns with the spatial distribution pattern of HuNoV enrichment in oyster digestive glands [76], but risk interpretation still requires consideration of genotype-specific differences. Strains such as GI.1 may exhibit higher enrichment efficiency and longer retention times in oysters, whereas the tissue kinetics of GII.4 strains may differ [43]. In samples containing mixed genotypes, a single total viral load may obscure the presence of highly retained or highly transmissible strains. In addition, viral loads in shellfish samples often approach the limit of detection [99], and minor losses during extraction, clarification, or concentration can influence the final result. Therefore, shellfish risk assessment should integrate tissue site, genotype composition, and method recovery efficiency.

#### 4.3.3. Decoupling Between Nucleic Acid Signals and Actual Infectivity

RT-qPCR remains the core tool for HuNoV detection, owing to its high sensitivity and strong basis for standardization. Within the ISO 15216 framework, detection sensitivity for GI and GII can reach low-copy levels [98]. However, RT-qPCR detects viral RNA, and RNA signals do not necessarily correspond to intact infectious particles [100]. Processing, storage, thermal treatment, or chemical disinfection may damage the viral capsid and eliminate infectivity while residual RNA remains amplifiable, leading to overestimation of risk.

Conversely, intact virions may be insufficiently recovered when they are strongly bound to matrix components, embedded within tissues, or protected within bacterial complexes, resulting in underestimation of risk. Aptamer-mediated enrichment of intact particles provides a potential complementary approach.

Aptamers such as AP4-2 and AP17-4 have been reported to block the binding of GII.4 and GII.17 virus-like particles (VLPs), rather than infectious HuNoV, to porcine gastric mucin (PGM) and to inhibit internalization-related processes in cell-based experimental models [96]. This approach may help reduce interference from free RNA, although its practical application in complex food samples remains insufficiently validated. Key uncertainties include aptamer stability, non-specific matrix interference, genotype breadth, and the operational robustness of intact-virion enrichment under routine food-testing conditions.

#### 4.3.4. Interference of Microecology and Matrix Components During Sample Preprocessing

The food microecological environment can alter the distribution behavior of HuNoVs during sample preprocessing. Virus–bacteria complexes, biofilm structures, and matrix components such as proteins, polysaccharides, and lipids may all influence the fate of viral particles during centrifugation, filtration, precipitation, elution, and lysis. If virions co-sediment with bacterial debris or biofilm matrices, clarification steps may unintentionally remove target particles. If virions are embedded within EPS or complex organic matter, lysis and nucleic acid release may also be reduced.

Such interference can lead to fluctuations in Ct values, and conventional process controls may not fully capture the specific binding behavior of HuNoVs within real food matrices. For example, process control viruses such as Mengovirus can monitor overall extraction efficiency [98,101], but may not adequately mimic the high-affinity interactions between HuNoVs and HBGAs, HBGA-like glycans, or bacterial envelope components. Accordingly, detection results from samples with high microbial loads or complex processing matrices should be interpreted together with matrix properties and the risk of preprocessing-associated viral loss. The matrix-specific retention mechanisms and their implications for food safety control discussed in this section are summarized in Table 1.

## 5. Future Perspectives

Food safety on HuNoV research has progressively evolved from host receptor recognition to the discovery of food matrix ligands, and further toward an understanding of complex ecological interactions. Early studies primarily relied on HBGAs to explain host susceptibility and genotype-dependent differences. Subsequently, the introduction of VLPs, P-particles, HIEs, bacterial cell surface display systems, and structural biology tools enabled researchers to dissect virus–ligand interactions despite the lack of a robust cultivation system. In recent years, the identification of plant-derived glycans, shellfish tissue ligands, and bacterial envelope components has further advanced HuNoV research into a new stage centered on the joint effects of food matrices and microecology. A central task for future research will be to translate these dispersed molecular findings into a systematic framework capable of explaining real-world foodborne transmission risks.

### 5.1. From Single Receptor/Ligand Recognition to Multi-Ligand Repertoires

HuNoV research was initially centered on HBGAs, a direction that laid the foundation for understanding secretor status, host susceptibility, and the predominance of epidemic strains. As the recognition patterns of GI and GII genotypes were gradually resolved, receptor/ligand-focused studies also began to reveal the pronounced structural adaptability and evolutionary flexibility of HuNoVs. With the subsequent identification of non-HBGA glycans, small-molecule ligands, plant-derived glycans, shellfish proteinaceous ligands, and bacterial surface components, the scope of HuNoV ligand recognition has expanded from a single host receptor paradigm to a broader range of viral binding interfaces.

As discussed above, HuNoV binding within food matrices is likely influenced by multiple ligand classes, including diverse glycans, proteinaceous ligands, and microbial components. In fresh produce, plant-derived glycans may contribute to viral attachment and retention, together with surface adsorption and tissue internalization processes. In shellfish, HBGA-like glycans and additional proteinaceous ligands have each been implicated in tissue-specific enrichment. Bacteria-related studies further suggest that microbiota-associated glycan analogs, envelope components, and extracellular matrices may modulate viral attachment, stability, and persistence across food environments. This multi-ligand perspective highlights the need for future studies on norovirus transmission, control strategies, and detection methods to move beyond isolated receptor- or ligand-centered analyses and to evaluate HuNoV persistence in the context of matrix-specific interactions within complex food ecosystems. At present, the evolutionary significance of such food-matrix-associated interactions remains less clearly defined than that of host-associated HBGA recognition, which is more directly linked to susceptibility and epidemic predominance. Because HuNoV does not replicate in foods, matrix-associated ligands are unlikely to constitute direct selective drivers in the same manner as host or immune pressures. Nevertheless, by influencing persistence and transmission opportunity, they may still contribute indirectly to the ecological context in which successful variants spread.

### 5.2. From Static Contamination Events to Dynamic Virus–Matrix Interaction Processes

Historically, food virology research often regarded the presence of HuNoV in foods as a relatively static contamination event, focusing mainly on whether the virus could bind a specific ligand, how strong the binding affinity was, and which amino acid residues participated in recognition. With the development of AFM, STD-NMR, molecular dynamics simulations, AlphaFold3, and related approaches, researchers now have increasing opportunities to observe conformational adjustments of the HuNoV capsid, changes in mechanical stability, and genotype-dependent differences during ligand engagement. These findings suggest that virus-ligand interactions should be understood as continuously changing and highly environment-dependent dynamic processes.

Such dynamic processes are jointly regulated by the physicochemical properties of food matrices, viral adaptive changes, host physiological status, and microbiota composition. For example, the same strain may exhibit different attachment efficiencies, tissue retention capacities, and environmental stability across distinct food matrices. The filter-feeding status of shellfish, the extent of plant tissue damage, and the composition of the food microecology may all alter the trajectory from contamination, attachment, internalization, and enrichment to eventual transmission. Accordingly, dynamic virus–matrix interaction mechanisms help explain why the same strain can display different transmission behaviors in different food systems. Future research may benefit from systems ecology-based approaches and multifactorial risk assessment models that more accurately reflect the dynamic transmission patterns of HuNoVs within global food trade chains.

### 5.3. From Model Systems to Multi-Scale Integration in Real Food Scenarios

A central limitation in current HuNoV research is the clear scale gap between molecular-level mechanistic resolution and the behavior of viruses within complex food matrices. The establishment of HIEs and zebrafish larval models has partially overcome the long-standing cultivation bottleneck of HuNoVs and has provided important platforms for studying infection mechanisms, genotype-dependent differences, and intervention efficacy. Nevertheless, viral replication in existing models remains limited; for example, replication titers in some systems reach approximately 10^3^–10^5^ PFU/mL, which is substantially lower than the high shedding levels observed after human infection, where fecal samples may contain 10^6^–10^9^ viral particles per gram. This discrepancy suggests that real infection and transmission processes may be regulated by auxiliary factors and ecological conditions that extend beyond single-receptor engagement.

At the same time, many ligand-binding experiments and computational studies are still conducted using purified ligands, recombinant proteins, VLPs/P-particles, or simplified buffer systems. These models can precisely resolve virus–ligand binding modes, key residues, and affinity differences, but they cannot fully reconstruct the substantial heterogeneity of real food systems. Actual food matrices contain diverse glycans, proteins, and lipids and are continuously influenced by processing, storage, temperature, pH, ionic strength, and microbiota dynamics. Therefore, strong binding at the molecular level does not necessarily translate into stable retention in real food scenarios, and blocking or inactivation effects observed in laboratory models may change substantially within complex matrices.

This conceptual gap is particularly evident in cross-matrix transmission studies. Molecular simulations can predict, with relatively high precision, the binding mode between HuNoV and lettuce-derived glycans, yet they cannot fully reproduce the dynamic process by which the virus enters plant tissues through root uptake, stomatal openings, or tissue damage. Similarly, the formation of virus–bacteria complexes can markedly enhance HuNoV tolerance to acute thermal stress and chlorine disinfection, while this microbiota-mediated stabilization remains difficult to fully replicate in standardized HIEs or simplified inactivation models. Consequently, intervention effects observed in the laboratory may be substantially weakened in real transmission scenarios.

Future HuNoV food safety research should therefore shift toward multiscale integration by coupling molecular interaction analysis, food contamination models, complex microbiota systems, and infectivity assessment platforms. Structural biology and computational modeling can identify key ligands and binding interfaces; real food models can validate viral attachment, enrichment, and response to decontamination within matrices; and HIEs or zebrafish models can further evaluate whether these matrix-associated states affect viral infectivity. By connecting evidence across these scales, future studies will be better positioned to explain HuNoV transmission risks in real food chains and to provide a reliable basis for precision detection and mechanism-driven intervention.

## 6. Conclusions

HuNoV foodborne transmission is not a simple process of passive viral contamination and residual persistence within food matrices, but rather a complex outcome shaped by viral structural features, ligand recognition, food matrix properties, and the microbial environment. The studies reviewed herein indicate that, beyond canonical HBGAs, HuNoVs may engage a broad range of candidate binding interfaces, including HBGA-like glycans, non-canonical glycans, plant-derived glycans, shellfish tissue glycans and proteinaceous ligands, as well as bacterial envelope components. These interactions may influence viral attachment and enrichment in foods, as well as environmental stability, detection recovery efficiency, and susceptibility to decontamination. Accordingly, understanding the physical and biological states in which HuNoVs persist within different food systems is important for explaining their continued transmission risk.

Building on this recognition, future HuNoV food safety research should more closely connect molecular mechanisms with real food scenarios. Conventional decontamination, standardized detection, and risk assessment methods remain important foundations for food safety control, but their efficacy and interpretation should be re-examined in light of matrix-specific virus-associated interactions. Continued investigation of matrix-specific ligand repertoires, dynamic virus–matrix processes, and multiscale model integration will help establish more biologically relevant detection systems and more precise intervention strategies, thereby improving the prediction and control of HuNoV foodborne transmission risks.

## Figures and Tables

**Table 1:** Mechanism-informed framework linking HuNoV adsorption and persistence in food matrices to control challenges and intervention strategies.

Food/Matrix Interface	Major Adsorption or Retention Mechanism	Control Challenge	Mechanism-Matched Intervention Strategy
Lettuce/leafy vegetable surface: non-specific initial adsorption	Initial adhesion is mainly driven by electrostatic and other physicochemical interactions.	Removal efficiency varies with matrix properties, ionic conditions, and genotype.	Optimize washing and pretreatment conditions to reduce initial surface attachment.
Lettuce/leafy vegetable internal tissues: tissue internalization	Entry through roots, stomata, or damaged tissues leads to protected internal persistence.	Internalized virions are poorly removed by surface washing or chlorination.	Reduce pre-harvest contamination and tissue damage; apply multi-barrier prevention.
Lettuce/leafy vegetable glycan ligands: specific molecular recognition	Lettuce H2N2F2-like glycans can bind P-domain pockets of prevalent genotypes.	High-affinity glycan binding limits elution, removal, and competitive blocking.	Develop P-domain-targeting blockers such as soluble glycans, glycoproteins, or aptamers.
Oyster: HBGA-like glycan-mediated tissue retention	HBGA-like A/H glycans, enriched in the digestive gland, promote tissue retention.	Strong digestive-gland binding reduces the effectiveness of depuration.	Optimize depuration conditions and explore glycan-competitive dissociation strategies.
Oyster: proteinaceous ligand-mediated genotype-specific binding	oHSP70, oTNF, and oIFT contribute to genotype-specific tissue binding.	Differential ligand binding may drive genotype-dependent accumulation and persistence.	Adopt genotype-aware depuration and explore interventions targeting key proteinaceous ligands.
Bacterial and microecological interfaces associated with lettuce and oysters	HBGA-like glycans, LPS, EPS, PG, and biofilms promote attachment and capsid stabilization.	Virus-bacterium complexes reduce disinfectant access and enhance stress tolerance.	Combine antibiofilm or matrix-disrupting pretreatments with conventional inactivation.

## Data Availability

No new data were created or analyzed in this study.

## Abbreviations

The following abbreviations are used in this manuscript:

AGE	Acute gastroenteritis
BPs	Binding pockets
EPS	Extracellular polymeric substances
FUTs	Fucosyltransferases
HBGAs	Histo-blood group antigens
HuNoV	Human norovirus
GI	Genogroup I
GII	Genogroup II
HIEs	Human intestinal enteroids
oHSP70	oyster heat shock protein 70
oIFT	oyster intraflagellar transport protein
oTNF	oyster tumor necrosis factor
VLPs	Virus-like particles
